# Effects of Sodium Benzoate, a D-Amino Acid Oxidase Inhibitor, on Perceived Stress and Cognitive Function Among Patients With Late-Life Depression: A Randomized, Double-Blind, Sertraline- and Placebo-Controlled Trial

**DOI:** 10.1093/ijnp/pyac006

**Published:** 2022-01-12

**Authors:** Chieh-Hsin Lin, Shi-Heng Wang, Hsien-Yuan Lane

**Affiliations:** Department of Psychiatry, Kaohsiung Chang Gung Memorial Hospital, Chang Gung University College of Medicine, Kaohsiung, Taiwan; Graduate Institute of Biomedical Sciences, China Medical University, Taichung, Taiwan; School of Medicine, Chang Gung University, Taoyuan, Taiwan; Department of Occupational Safety and Health; Department of Psychiatry and Brain Disease Research Center, China Medical University Hospital, Taichung, Taiwan; Department of Psychology, College of Medical and Health Sciences, Asia University, Taichung, Taiwan

**Keywords:** Cognition, late, life depression, N-methyl-D-aspartate, perceive stress, sodium benzoate

## Abstract

**Background:**

Compared with adults with depression in the general population, elderly depressive patients are prone to poor treatment response, more side effects, and early withdrawal with current antidepressants (which principally modulate monoamines). Whether N-methyl-D-aspartate receptor enhancement can benefit treatment of late-life depression deserves study. This study aims to compare sodium benzoate (a D-amino acid oxidase inhibitor and an indirect N-methyl-D-aspartate receptor enhancer), sertraline (a selective serotonin reuptake inhibitor), and placebo in the treatment of late-life depression.

**Methods:**

In this randomized, double-blind trial, 117 patients with major depressive disorder aged 55 years or older received 8-week treatment of 250–1500 mg/d of sodium benzoate, 25–150 mg/d of sertraline, or placebo in 2 medical centers. The primary outcome measures were Hamilton Depression Rating Scale and Perceived Stress Scale scores.

**Results:**

Three treatments similarly decreased clinicians-rated Hamilton Depression Rating Scale scores. Compared with placebo, sodium benzoate but not sertraline substantially improved Perceived Stress Scale scores and cognitive function. Sertraline, but not benzoate, significantly reduced self-report Geriatric Depression Scale scores. Benzoate and placebo showed similar safety profiles, while sertraline was more likely to raise low-density lipoprotein than benzoate and placebo. Benzoate-treated patients were less likely to drop out than sertraline or placebo recipients.

**Conclusions:**

Sertraline can reduce subjective depressive symptoms, while benzoate can decrease perceived stress, improve cognitive function, and enhance treatment adherence in late-life depression patients. The results show promise for D-amino acid oxidase inhibition as a novel approach for perceived stress and cognitive decline among patients with late-life depression.

**Trial Registration:**

ClinicalTrials.gov Identifier: NCT03414931. Registered January 2016.

**Significance Statement**

Efficacy of antidepressants for late-life depression remains uncertain. Whether N-methyl-D-aspartate receptor (NMDAR) enhancement can benefit treatment of late-life depression deserves study. In this randomized, double-blind, 8-week trial in 117 patients with late-life depression, sertraline (a selective serotonin reuptake inhibitor) reduced depressive symptoms, while sodium benzoate (a D-amino acid oxidase [DAAO] inhibitor and an indirect NMDAR enhancer) decreased perceived stress, improved cognitive function, and increased treatment adherence of the patients. Benzoate appeared safer than sertraline. The results show promise for DAAO inhibition as a novel approach for perceived stress and cognitive decline among patients with late-life depression.

## Introduction

Major depressive disorder (MDD) is a severe and common mental disorder in the elderly (Steffens et al., 2000; Chong et al., 2001; Kok and Reynolds, 2017). Approximately 10%–15% of the elderly in the community have some depressive symptoms (Copeland et al., 1999; Kok and Reynolds, 2017). Depression in older adults is associated with perceived stress (Deng et al., 2018; Bickford et al., 2020) and cognitive deficits (Nebes et al., 2003; Butters et al., 2004; Mecocci et al., 2004; Salazar-Villanea et al., 2015). Moreover, late-life depression is commonly comorbid with medical illnesses and is also a risk factor for other diseases such as dementia and coronary artery disease (Mecocci et al., 2004). Due to its excess disability, morbidity, and mortality (Miu and Chan, 2011; Deng et al., 2018), late-life depression has become a major public health problem, especially in the rapid-aging society (Lin et al., 2014b; Taylor, 2014; Deng et al., 2018).

Most of the current antidepressants are based on the monoamine hypothesis; however, approximately 30%–60% of patients with MDD fail to recover (Keller et al., 1992; Riihimaki et al., 2014). In addition, only one-half of elder people respond to pharmacological treatments (Cooper et al., 2011). In late-life depression, the response rate to antidepressants is lower compared with depression in younger patients, but the placebo response rate is similar (Tedeschini et al., 2011; Alexopoulos, 2019); the efficacy comparison between antidepressants and placebo in late-life depression is inconsistent among studies (Tedeschini et al., 2011; Kok et al., 2012).

Further, the antidepressant (sertraline and mirtazapine) group had more adverse reactions than the placebo group in elderly patients (Banerjee et al., 2011), therefore hampering medication adherence (Zivin and Kales, 2008). Moreover, mood improvement was not necessarily associated with cognitive improvement after antidepressant treatment for geriatric depressed patients (Butters et al., 2000; Devanand et al., 2003; Nebes et al., 2003; Morimoto and Alexopoulos, 2013). Abnormal speed of processing, working memory, episodic memory, and executive functions (such as reasoning and problem solving) persist after remission of mood symptoms in many patients with late-life depression (Butters et al., 2000; Nebes et al., 2003; Aizenstein et al., 2009; Morimoto and Alexopoulos, 2013). Hence, there is an urgent need to develop novel therapies with broader efficacy profiles including cognitive-enhancing activity for late-life depression.

Glutamate is the most abundant amino acid neurotransmitter in mammalian brain. N-methyl-D-aspartate receptor (NMDAR), a subtype of ionotropic glutamate receptor, plays an important role in modulating mood and cognition (Krystal et al., 1994; Mathews et al., 2012; Guercio and Panizzutti, 2018; Chang et al., 2019). Depression may have complex neural substrates in that both up- and downregulation of NMDAR function are involved (Huang et al., 2017); and both antagonists (such as ketamine, esketamine, and arketamine) ( Krystal et al., 2019; Turner, 2019; Berman et al., 2000; Hashimoto, 2020) and agonists (such as D-serine, sarcosine, and sodium benzoate) can be antidepressant therapies (Lai et al., 2012; Huang et al., 2013; Levin et al., 2015). Of note, NMDAR antagonists and agonists shared a common mechanism in treating depression by activating AMPA receptors (Chen et al., 2015; Wei et al., 2017).

Importantly, the efficacy and safety of NMDAR modulators for treatment of late-life depression remain uncertain (Hsieh, 2019). Among the NMDAR modulators that can improve depressive mood, sodium benzoate, a D-amino acid oxidase inhibitor, has been found to be able to improve cognitive function of patients with schizophrenia (Lane et al., 2013; Lin et al., 2017) or early-phase Alzheimer’s disease (Lin et al., 2014a). Whether it can also benefit cognitive function of late-life depression patients deserves study. This study aimed to compare sodium benzoate, sertraline (an selective serotonin reuptake inhibitor [SSRI]), and placebo for the treatment of late-life depression.

## METHODS

This was an 8-week, randomized, double-blind, sertraline- and placebo-controlled trial, which was registered at ClinicalTrials.gov (https://clinicaltrials.gov/ct2/show/NCT03414931), conducted by 2 major medical centers, Kaohsiung Chang Gung Memorial Hospital, Kaohsiung, and China Medical University Hospital, Taichung, in Taiwan, and approved by the Chang Gung Medical Foundation Institutional Review Board (no. 201200365A3C506) and China Medical University Hospital Institutional Review Board (no. DMR101-IRB2-100). Written informed consent was obtained from all participants.

### Participants

Patients were evaluated by research psychiatrists and enrolled into this study if they (1) were aged 55 years or older; (2) satisfied DSM-IV criteria for MDD (American Psychiatric Association, 1994); (3) had a minimum baseline total score of 18 on the 17-item Hamilton Rating Scale for Depression (HAMD); (4) had a minimum baseline score of 20 on the Mini-Mental State Examination (Folstein et al., 1975); and (5) were free of psychotropic drugs for at least 2 weeks.

Exclusionary criteria included (1) current substance abuse or history of substance dependence in the past 6 months; (2) history of epilepsy, head trauma, stroke, or other serious medical or neurological illness, (3) bipolar depression, schizophrenia, or other psychotic disorder; (4) pathologically abnormal findings in laboratory assessments, including blood routine and biochemistry test; (5) moderate-severe suicidal risks; (6) severe cognitive impairment; (7) initiating or stopping formal psychotherapy within 6 weeks prior to enrollment; (8) history of poor response or severe adverse reaction to SSRIs or other antidepressants; (9) history of previously received electroconvulsive therapy; (10) use of depot antipsychotics in the past 6 months; and (11) inability to follow protocol.

### Study Design

All patients were randomly assigned to 3 groups: placebo, sertraline (25–150 mg/d), or sodium benzoate (250–1500 mg/d). Patients were randomized in clusters of 6 participants through a computer-generated randomization table to receive placebo, sertraline, or sodium benzoate in a 1:1:1 ratio. To ensure concealment of the randomization assignment, medication was provided with supply of identical-appearing capsules of placebo, sertraline (25 mg per capsule), or benzoate (250 mg per capsule). The dose was started at 1–2 capsules per day (1 capsule once or twice daily) in the first 2 weeks, then increased or decreased by 1–2 capsules per day every 2 weeks (from the beginning of the third, fifth, or seventh week), if clinically indicated. Study medications were given once daily when the dose was at 1 capsule per day or given twice daily when the dose was at 2 capsules or higher per day. The dose range (250–1500 mg/d) of benzoate was the same as that used in the study exploring the efficacy of benzoate for elderly patients with behavioral and psychological symptoms of dementia (BPSD); at these doses, benzoate showed excellent safety profiles (Lin et al., 2019; Lin et al., 2020).

Patients, caregivers, and investigators, except the investigational pharmacist, were all blind to the assignment. Patient medical adherence and safety were closely monitored by caregivers and research physicians, and pill-counting was monitored by the study staff.

During the study, limited use of benzodiazepines (up to 4 mg/d lorazepam) was allowed as concomitant medication for agitation or insomnia. In case of side-effect intolerance or clinical worsening, the patients could be withdrawn earlier.

### Assessments

The primary outcome measures were clinical improvement assessed by clinician-rated HAMD total score (Hamilton, 1960) and self-reported Perceived Stress Scale (PSS) (Cohen et al., 1983).

The secondary outcome measures included dropout rate and clinical improvements measured by self-report Geriatric Depression Scale (GDS) (Sheikh, 1986), clinician-rated Clinical Global Impression-Severity (CGI) (Guy, 1976), self-reported Beck’s Suicide Scale (BSS) (Beck et al., 1979), and cognitive function composed of 4 domains: (1) Wechsler Adult Intelligence Scale-III Digit Symbol-Coding to assess speed of processing; (2) Wechsler Adult Intelligence Scale-III Digit Span to assess working memory; (3) Wechsler Memory Scale-III Logical Memory Test to assess episodic memory (Wechsler, 1997); and (4) Wechsler Intelligence Scale for Children-III Maze to measure reasoning and problem solving (Wechsler, 1991). To minimize the learning effect, cognitive function was measured at baseline (week 0) and at endpoint, while other outcome measures were administered every 2 weeks (at weeks 0, 2, 4, 6, and 8).

The cognition composite score was calculated by the average of the T scores of the 4 cognitive domains (speed of processing, working memory, episodic memory, and reasoning and problem solving). The raw score of each cognitive domain was standardized to a T score with a mean of 50 and an SD of 10 for making each test comparative.

Systemic adverse effects were examined every 2 weeks (at weeks 0, 2, 4, 6, and 8) by routine physical and neurological examinations and the Udvalg for Kliniske Undersogelser Side-effects Rating Scale (Lingjaerde et al., 1987). Routine laboratory tests, including CBC and biochemistry, were checked at baseline and endpoint.

Among clinical rating scales, PSS (Cohen et al., 1983), GDS (Sheikh, 1986), and BSS (Beck et al., 1979) were designed to be self-report rating scales. HAMD (Hamilton, 1960) and CGI (Guy, 1976) were designed to be rated by clinicians and in the current study were performed by the research psychiatrists who were trained and experienced in the rating scales. Inter-rater reliability was analyzed with the ANOVA test. Only raters who reached the intra-class correlation coefficients of *>*0.90 during prestudy training were allowed to rate the study patients. To maintain high inter-rater reliability and to prevent rater drift, raters met at least once per quarter for training and reliability retesting. To minimize inter-rater variability, each individual patient was assessed by the same raters throughout the trial.

### Data Analysis

Chi-square test (or Fisher’s exact test) was used to compare differences of categorical variables and 1-way ANOVA (or Kruskal-Wallis test if the distribution was not normal) for continuous variables among 3 treatment groups. To compare the changes from baseline in repeated-measure assessments, we used the generalized estimating equation method’s multiple linear regression models with treatment, visit, and treatment-visit interaction terms after adjusting the baseline value of the outcome measure. The working correlation matrix was specified as autoregressive of order 1.

All data were analyzed by SPSS version 22.0 (IBM Corp., Armonk, NY, USA). All *P* values for clinical measures were based on 2-tailed tests with a significance level of .05.

## RESULTS

### Patient Disposition and Characteristics

**Figure 1.**
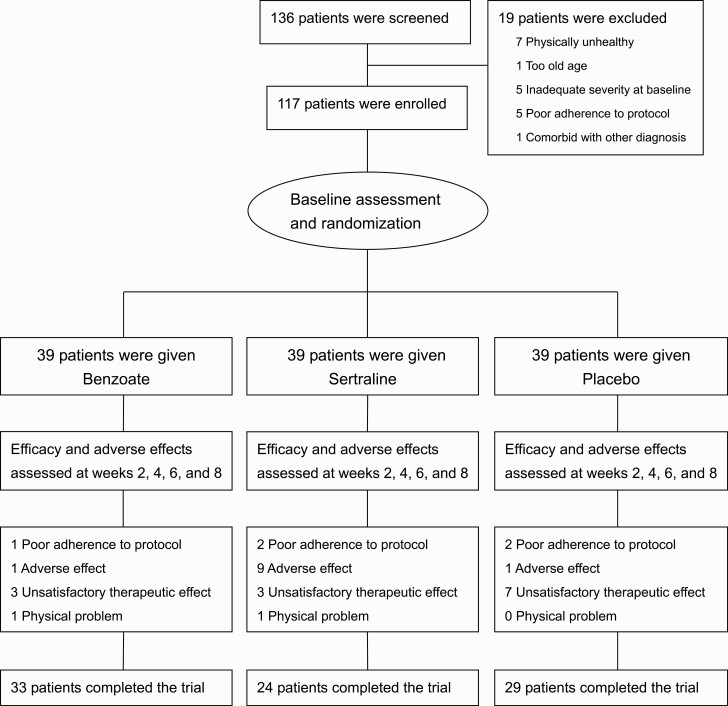
Flow diagram and disposition of the 3 treatment groups.

A total of 136 patients were screened. Nineteen of the patients were excluded due to screening failure. A total of 117 patients were eligible and randomly allocated to 3 treatment groups, with 39 patients in each group (Fig. 1).

Of the 117 eligible patients, gender, education level, body mass index, various illness severity (in HAMD, PSS, GDS, CGI, BSS), and Mini-Mental State Examination score at baseline were similar among the 3 groups, while the placebo group appeared older (*P* = .040) (Table 1).

**Table 1.** Baseline Demographic Characteristics of the Sodium Benzoate, Sertraline, or Placebo Treatment Groups

	Treatment Groups			P
	Benzoate (n = 39)	Sertraline (n = 39)	Placebo (n = 39)	
Gender, female, n (%)	27 (69.2)	34 (87.2)	29 (74.4)	.15^*a*^
Age, y, mean (SD)	66.4 (8.1)	66.9 (7.2)	70.5 (7.3)	.040^*c*^
Education, y, mean (SD)	7.5 (4.9)	6.7 (4.5)	6.7 (3.9)	.62^*c*^
BMI, mean (SD)	22.9 (3.2)	23.7 (4.5)	24.2 (3.7)	.44^*c*^
Dose at endpoint, mg/d, mean (SD)	769.2 (239.1)	66.7 (30.5)	NA	
HAMD score, mean (SD)	26.1 (5.1)	27.0 (5.2)	25.4 (6.0)	.48^*b*^
PSS score, mean (SD)	36.2 (5.9)	36.3 (4.5)	35.4 (5.3)	.70^*b*^
GDS score, mean (SD)	10.7 (3.5)	11.5 (3.0)	10.5 (2.7)	.30^*c*^
CGI, mean (SD)	3.8 (0.5)	4.0 (0.5)	3.8 (0.5)	.25^*c*^
BSS score, mean (SD)	7.1 (6.5)	6.8 (6.1)	6.8 (6.4)	.97^*c*^
MMSE score, mean (SD)	25.3 (3.2)	24.6 (4.6)	25.4 (3.6)	.80^*b*^

Abbreviations: BMI, body mass index; BSS, Beck’s Suicide Scale; CGI, Clinical Global Impression-Severity; GDS, Geriatric Depression Scale; HAMD, 17-item Hamilton Rating Scale for Depression; MMSE, Mini-Mental State Examination; PSS, Perceived Stress Scale.

^*a*^Chi-square test.

^*b*^ANOVA test.

^*c*^Kruskal-Wallis test.

Finally, the mean endpoint dose of sodium benzoate was 769.2 ± 239.1 (SD) mg/d and that of sertraline was 66.7 ± 30.5 mg/d (Table 1). Thirty-three (84.6%) patients in the benzoate group, 24 (61.5%) in the sertraline group, and 29 (74.4%) in the placebo group completed the 8-week trial (Fig. 1). Of the 6 benzoate receivers who withdrew earlier, 3 were due to unsatisfactory therapeutic effect. Of the 15 sertraline receivers who withdrew, 9 were due to adverse effects. Of the 10 placebo recipients who withdrew, 7 were due to unsatisfactory therapeutic effect (Fig. 1). It appears that sertraline receivers tended to drop out earlier due to adverse effects, and placebo-treated patients tended to withdraw with poor treatment response.

### Primary Outcome Measures

Regarding psychiatrist-rated depression measurement, sodium benzoate treatment did not differ significantly from sertraline or placebo in decreasing HAMD scores at weeks 2–8 and endpoint (Table 2). On the other hand, sertraline and placebo also did not differ in changing HAMD throughout the treatment period and at endpoint (*P* ranging from .13 to .75; detailed statistics are not shown).

**Table 2.** Results of Measures of Primary Outcomes Over the 8-Week Treatment Using the GEE Method, Which Simultaneously Compared the 3 Treatment Groups Using a Single Analysis

Scale	Benzoate	Sertraline	Placebo	Sertraline vs benzoate	Placebo vs benzoate
	Mean ± SD (n)	Mean ± SD (n)	Mean ± SD (n)	Estimate, SE, Z (*P* value)	Estimate, SE, Z (*P* value)
HAMD					
Baseline	26.1 ± 5.1 (39)	27.0 ± 5.2 (39)	25.4 ± 6.9 (39)	0.78, 1.11, 0.70 (.49)^*a*^	−0.67, 1.06, −0.63 (.53)^*a*^
Week 2	23.4 ± 6.5 (37)	23.8 ± 5.4 (34)	22.6 ± 6.3 (38)	−0.28, 0.86, −0.33 (.74)^*b*^	−0.04, 0.87, −0.04 (.97)^*b*^
Week 4	22.4 ± 6.0 (37)	22.0 ± 5.4 (28)	21.4 ± 7.2 (32)	−0.85, 1.13, −0.75 (.45)^*b*^	0.38, 1.10, 0.34 (.73)^*b*^
Week 6	20.7 ± 5.4 (35)	20.8 ± 5.7 (26)	18.9 ± 6.9 (29)	−0.57, 1.22, −0.47 (.64)^*b*^	0.09, 1.32, 0.06 (.95)^*b*^
Week 8	19.9 ± 6.0 (34)	18.7 ± 6.3 (24)	19.3 ± 7.2 (29)	−1.12, 1.32, −0.85 (.40)^*b*^	1.02, 1.41, 0.73 (.47)^*b*^
Endpoint	20.9 ± 6.9 (37)	20.8 ± 7.2 (34)	21.3 ± 8.4 (38)	−0.88, 1.32, −0.67 (.51)^*b*^	1.11, 1.40, 0.80 (.43)^*b*^
PSS					
Baseline	36.2 ± 5.9 (39)	36.3 ± 4.5 (39)	35.4 ± 5.3 (39)	0.05, 1.14, 0.04 (.97)^*a*^	−0.98, 1.19, −0.82 (.42)^*a*^
Week 2	36.5 ± 6.4 (37)	34.3 ± 4.8 (34)	34.5 ± 5.9 (38)	−1.64, 1.22, −1.35 (.18)^*a*^	−0.76, 1.16, −0.66 (.51)^*b*^
Week 4	34.8 ± 6.9 (37)	35.3 ± 3.8 (28)	35.2 ± 5.2 (32)	0.55, 1.28, 0.43 (.67)^*b*^	1.55, 1.13, 1.37 (.17)^*b*^
Week 6	34.7 ± 7.5 (35)	35.0 ± 3.7 (26)	34.0 ± 6.0 (29)	−0.26, 1.46, −0.18 (.86)^*b*^	−0.10, 1.55, −0.06 (.95)^*b*^
Week 8	30.9 ± 8.4 (33)	34.2 ± 3.8 (24)	34.4 ± 5.6 (29)	2.16, 1.74, 1.24 (.22)^*b*^	3.34, 1.68, 1.99 (.047)^*b*^
Endpoint	32.8 ± 9.9 (37)	34.5 ± 4.5 (34)	34.8 ± 5.8 (38)	1.84, 1.76, 1.05 (.30)^*b*^	3.07, 1.69, 1.82 (.07)^*b*^

Abbreviations: GEE, Generalized Estimating Equation; HAMD, 17-item Hamilton Rating Scale for Depression; PSS, Perceived Stress Scale; SE, standard error.

^*a*^Comparison was based on the average of the total score.

^*b*^Comparisons was based on the changes from the baseline in average of total score. Estimate is the coefficient of treatment and treatment-visit interaction term in the GEE method’s multiple linear regression model by specifying the working correlation matrix as autoregressive of order 1, AR (1). *P* values were based on 2-tailed tests.

As for perceived stress, benzoate excelled placebo in reducing PSS scores at week 8 (Table 2), while benzoate and sertraline did not differ significantly in altering PSS (Table 2). Further, sertraline and placebo did not differ in altering PSS at all visits and endpoint (*P* ranging from .29 to .90).

### Secondary Outcome Measures

Regarding self-report depression assessment, benzoate did not differ significantly from sertraline or placebo in diminishing GDS scores (Table 3). However, sertraline was better than placebo in lessening GDS scores at week 4, week 8, and endpoint (sertraline vs placebo, week 4, estimate = −1.45, SE = 0.71, Z = 2.03, *P* = .042; week 8, estimate = −1.61, SE = 0.79, *Z = *2.04, *P* = .041; endpoint, estimate = −1.58, SE = 0.77, *Z* = 2.05, *P* = .040).

**Table 3.** Results of Measures of Secondary Outcomes Over the 8-Week Treatment Using the GEE method, Which Simultaneously Compared the 3 Treatment Groups Using a Single Analysis

Scale	Benzoate	Sertraline	Placebo	Sertraline vs benzoate	Placebo vs benzoate
	Mean ± SD (N)	Mean ± SD (N)	Mean ± SD (N)	Estimate, SE, Z (*P* value)	Estimate, SE, Z (*P* value)
GDS					
Baseline	10.3 ± 3.1 (39)	11.2 ± 2.6 (39)	10.5 ± 2.7 (39)	0.82, 0.62, 1.34 (.18)^*a*^	0.03, 0.60, 0.06 (.95)^*a*^
Week 2	9.8 ± 3.3 (37)	9.5 ± 2.9 (34)	9.5 ± 3.1 (38)	−1.03, 0.61, −1.69 (.09)^*b*^	−0.39, 0.61, −0.64 (.53)^*b*^
Week 4	9.2 ± 3.3 (37)	9.1 ± 3.2 (28)	9.6 ± 2.9 (32)	−0.65, 0.72, −0.90 (.37)^*b*^	0.80, 0.69, 1.15 (.25)^*b*^
Week 6	9.1 ± 3.4 (35)	8.9 ± 3.2 (26)	8.5 ± 3.0 (29)	−1.04, 0.80, −1.30 (.20)^*b*^	−0.12, 0.80, −0.15 (.88)^*b*^
Week 8	8.2 ± 3.8 (34)	7.7 ± 3.6 (24)	8.4 ± 2.8 (29)	−1.00, 0.89, −1.13 (.26)^*b*^	0.61, 0.81, 0.76 (.45)^*b*^
Endpoint	8.7 ± 4.0 (37)	8.6 ± 3.8 (34)	9.4 ± 3.3 (38)	−0.90, 0.87, −1.03 (.30)^*b*^	0.69, 0.81, 0.85 (.39)^*b*^
CGI					
Baseline	3.8 ± 0.5 (39)	4.0 ± 0.5 (39)	3.8 ± 0.5 (39)	0.18, 0.11, 1.61 (.11)^*a*^	0.02, 0.11, 0.20 (.84)^*a*^
Week 2	3.8 ± 0.5 (37)	3.8 ± 0.4 (34)	3.7 ± 0.5 (38)	−0.08, 0.12, −0.68 (.50)^*b*^	−0.01, 0.13, −0.11 (.92)^*b*^
Week 4	3.7 ± 0.6 (37)	3.6 ± 0.5 (28)	3.6 ± 0.6 (32)	−0.12, 0.15, −0.82 (.41)^*b*^	−0.09, 0.15, −0.62 (.54)^*b*^
Week 6	3.5 ± 0.6 (35)	3.5 ± 0.6 (26)	3.4 ± 0.6 (29)	−0.20, 0.16, −1.23 (.22)^*b*^	−0.16, 0.15, −1.03 (.30)^*b*^
Week 8	3.4 ± 0.6 (34)	3.3 ± 0.6 (24)	3.4 ± 0.8 (29)	−0.16, 0.16, −0.97 (.33)^*b*^	−0.03, 0.17, −0.16 (.87)^*b*^
Endpoint	3.5 ± 0.7 (37)	3.6 ± 0.7 (34)	3.5 ± 0.8 (38)	−0.13, 0.16, −0.78 (.43)^*b*^	−0.00, 0.17, −0.03 (.98)^*b*^
BSS					
Baseline	7.1 ± 6.5 (39)	6.8 ± 6.1 (39)	6.8 ± 6.4 (39)	−0.24, 1.37, −0.17 (.86)^*a*^	−0.20, 1.34, −0.15 (.88)^*a*^
Week 2	6.7 ± 5.9 (37)	4.9 ± 4.3 (34)	4.8 ± 4.9 (38)	−0.69, 0.51, −1.35 (.18)^*b*^	−0.89, 0.54, −1.66 (.10)^*b*^
Week 4	5.9 ± 5.2 (37)	4.6 ± 3.2 (28)	4.5 ± 5.1 (32)	−0.93, 0.77, −1.20 (.23)^*b*^	−0.39, 0.67, −0.58 (.56)^*b*^
Week 6	5.0 ± 4.5 (35)	4.2 ± 3.0 (26)	3.4 ± 3.0 (29)	−0.63, 0.89, −0.70 (.48)^*b*^	−0.49, 0.82, −0.60 (.55)^*b*^
Week 8	4.2 ± 3.3 (34)	3.6 ± 2.9 (24)	3.9 ± 4.4 (29)	−0.58, 0.96, −0.60 (.55)^*b*^	0.37, 0.96, 0.39 (.70)^*b*^
Endpoint	5.0 ± 4.4 (37)	3.8 ± 3.2 (34)	4.8 ± 5.6 (38)	−0.65, 0.96, −0.68 (.50)^*b*^	0.30, 0.94, 0.32 (.75)^*b*^
Dropout rate	N (%)	N (%)	N (%)	*P* value	
Week 2	2/39 (5.1%)	9/39 (23.1%)	3/39 (7.7%)	(.031)^*c*^	
Week 4	2/39 (5.1%)	13/39 (33.3%)	10/39 (25.6%)	(.007)^*c*^	
Week 6	5/39 (12.8%)	15/39 (38.5%)	10/39 (25.6%)	(.035)^*c*^	
Week 8	6/39 (15.4%)	15/39 (38.5%)	10/39 (25.6%)	(.07)^*c*^	
Overall	6/39 (15.4%)	15/39 (38.5%)	10/39 (25.6%)	(.07)^*c*^	
Cognitive function^*e*^	Mean ± SD (N)	Mean ± SD (N)	Mean ± SD (N)	F (*P* value)	
Baseline	49.4 ± 7.0 (38)	50.5 ± 8.8 (37)	50.3 ± 5.4 (36)	0.22 (.80)^*d*^	
Endpoint	51.3 ± 7.7 (35)	49.1 ± 7.7 (31)	49.5 ± 6.6 (35)	0.82 (.44)^*d*^	
Difference	2.1 ± 5.3 (35)	−0.6 ± 4.7 (31)	−0.6 ± 4.1 (35)	3.60 (.031)^*d*^	

Abbreviations: BSS, Beck’s Suicide Scale; CGI, Clinical Global Impression-Severity; GDS, Geriatric Depression Scale; GEE, Generalized Estimating Equation; SE, standard error.

^*a*^Comparison was based on the average of the total score.

^*b*^Comparisons was based on the changes from the baseline in average of total score. Estimate is the coefficient of treatment and treatment-visit interaction term in the GEE method’s multiple linear regression model by specifying the working correlation matrix as autoregressive of order 1, AR(1). *P* values were based on 2-tailed tests.

^*c*^Chi-square test.

^*d*^ANOVA test.

^*e*^Global composite score. For assessing the global composite, an overall composite T score that included 4 domains: (1) Wechsler Adult Intelligence Scale (WAIS)-III Digit Symbol-Coding to assess speed of processing, (2) WAIS-III Digit Span to assess working memory, (3) Wechsler Memory Scale-III Logical Memory Test to assess episodic memory,^53^ and (4) Wechsler Intelligence Scale for Children-III Maze to measure reasoning and problem solving^54^) was calculated by standardizing the sum of T scores.

Regarding clinical global severity, benzoate treatment did not differ significantly from sertraline or placebo in lowering CGI scores (Table 3). Sertraline and placebo also did not differ in their performances in CGI decrement from baseline to endpoint (*P* ranging from .48 to .76).

Concerning suicidal severity, benzoate treatment did not differ significantly from sertraline or placebo in lowering BSS scores (Table 3). Sertraline and placebo were similar in their effects in BSS reduction throughout the study period (*P* ranging from .33 to .86).

Benzoate-treated patients were less likely to drop out than the other 2 treatment groups of patients at weeks 2, 4, and 6 (Table 3). The benzoate group also performed best among the 3 treatment groups in cognitive function after treatment (Table 3).

### Adverse Effects

Benzoate and placebo showed similar safety profiles, as measured by Udvalg for Kliniske Undersogelser Side-effects Rating Scale (Lingjaerde et al., 1987) (Table 4).

**Table 4.** Treatment-Emergent Adverse Events During the Study

	No. of Participants		
	Benzoate	Sertraline	Placebo
Concentration difficulties	2		1
Asthenia/increased fatigability		5	1
Sleepiness/sedation	4	2	2
Failing memory	1	1	1
Depression	6	3	7
Tension/inner unrest	2	3	4
Increased duration of sleep	2		3
Reduced duration of sleep	5	5	9
Increased dream activity		1	
Emotional indifference			1
Rigidity	1		1
Tremor		1	1
Akathisia		2	
Epileptic seizures			1
Parasthesias		1	
Reduced salivation			1
Nausea/vomiting	1	1	
Diarrhoea	1		
Constipation	2	1	
Orthostatic dizziness	2		
Palpitations/tachycardia	1	1	
Pruritus		1	
Total	30	28	33

Low-density lipoprotein (LDL) levels were more likely to increase among patients in the sertraline group than those in the other 2 groups (Table 5). The 3 groups showed similar changes in other blood parameters in CBC and biochemistry (not shown).

**Table 5.** Baseline and Endpoint Measures of LDL

	Treatment Groups						*P*
	Benzoate		Sertraline		Placebo		
	n	mean (SD)	n	mean (SD)	n	mean (SD)	
LDL (mg/dL)							
Baseline	39	104.6 (36.8)	39	96.8 (30.3)	39	117.9 (50.1)	.07^a^
Endpoint	34	104.7 (36.6)	30	105.1 (36.2)	34	111.0 (48.4)	.80^b^
Difference	34	–5.7 (18.0)	30	9.7 (20.8)	34	−8.1 (22.5)	.010 ^b^

Abbreviation: LDL, low-density lipoprotein.

^a^ANOVA test.

^b^Kruskal Wallis test.

## DISCUSSION

To our knowledge, this is the first study to test the efficacy and safety of an NMDAR enhancer in the treatment of late-life depression. In addition, to our knowledge it is also the first to compare an NMDAR enhancer with both an antidepressant and placebo in the treatment of MDD. The findings of this study suggest that a commonly used SSRI (sertraline) can reduce subjectively depression symptoms, while an NMDAR enhancer (sodium benzoate herein) can decrease perceived stress, improve cognitive function, and enhance treatment adherence in patients with late-life depression. In addition, benzoate and placebo appeared safer than sertraline in not raising LDL levels.

Antidepressant medications have been suggested to be less effective in geriatric populations (Tedeschini et al., 2011; Deng et al., 2018; Alexopoulos, 2019). In the current study, though sertraline, benzoate, and placebo showed similar effects in reducing depressive symptoms measured by the psychiatrist-rated scale (HAMD), sertraline surpassed benzoate and placebo in improving the score of the elderly patient-rated depression scale (GDS), perhaps suggesting that GDS, originally designed for evaluation of geriatric depression (Sheikh, 1986), may be more suitable for study in depressive elderly people. More studies are necessary to confirm this notion.

Earlier, another NMDAR enhancer, sarcosine, surpassed citalopram (another SSRI) in decreasing depressive symptoms of non-elderly depressive patients in a double-blind trial, which was not controlled by placebo (Huang et al., 2013). Though benzoate has been suggested to be the most potent NMDAR enhancer in the treatment of schizophrenia, including ultra-resistant (clozapine-resistant) schizophrenia (Lane et al., 2013; Lin et al., 2017; Harrison, 2018; Lin et al., 2018), the efficacy of sarcosine and other NMDAR modulators (enhancers or antagonists) for the treatment of depression in the elderly needs further studies (George et al., 2017; Hsieh MT, 2019; Lipsitz et al., 2021).

A substantial fraction of patients with late-life depression continue to experience cognitive deficits after antidepressant treatment (Butters et al., 2000; Devanand et al., 2003; Nebes et al., 2003; Morimoto and Alexopoulos, 2013). Of note, benzoate was the only treatment to improve patient cognitive function in the current study, implying that benzoate may be a cognitive enhancer. In addition, benzoate’s cognition-enhancing activity may have been primary (i.e., not secondary to antidepressant activity). Similarly, benzoate also benefited cognitive function of patients with schizophrenia, no matter whether it decreased their psychotic symptoms (Lane et al., 2013) or not (Lin et al., 2017). Further, benzoate improved cognitive function of patients with early-phase Alzheimer’s disease (Lin et al., 2014a) and a portion of patients with BPSD (Lin et al., 2019, 2020, 2021); it also altered brain activity as well as cognitive functions in individuals with mild cognitive impairment (Lane et al., 2021). More studies are needed to explore the potential of benzoate’s cognition-enhancing effects in neuropsychiatric disorders.

Benzoate, but not sertraline, was able to decrease perceived stress (Table 2). Perceived stress, which seems higher in the elderly than in younger adults (Osmanovic-Thunstrom et al., 2015), is among the top 5 psychological health problems of the elderly population (Payne et al., 2014). The development of depression usually follows the perception of stress (Pizzagalli et al., 2007; Banjongrewadee et al., 2020). Perceived stress frequently leads to severe physical and mental consequences, including cardiovascular disorders and diseases related to poor immunological function, sleep problems, anxiety, and depression (Keller et al., 2012; Rueggeberg et al., 2012; Vasunilashorn et al., 2013; O’Neal et al., 2015; Banjongrewadee et al., 2020). Previous studies showed that NMDAR agonists differed in their roles in modulating stress reactivity: while reducing brain D-serine improved stress resilience (Dong et al., 2018), VU0410120, a glycine type 1 transporter inhibitor (and thereby an indirect NMDAR agonist), improved stress reactivity and sociability in a mouse study (Burket et al., 2020). Whether benzoate (also an indirect NMDAR agonist) exerts its effects on perceived stress in late-life depression via NMDAR activation deserves further studies. Other possible mechanisms included inflammation. Perceive stress has been associated with greater inflammation in healthy or obese adults (Jain et al., 2007; Casaletto et al., 2018; Zou et al., 2020), and sodium benzoate has been reported to reduce microglial and astroglial inflammatory responses (Brahmachari et al., 2009).

Late-life depression is associated with poor outcomes (such as disability and increased all-cause mortality) and a high risk of recurrence, particularly in those patients with poor treatment adherence (Taylor, 2014; Deng et al., 2018). In the current study, benzoate receivers displayed better treatment adherence with fewer dropouts than sertraline- and placebo-treated patients (Fig. 1; Table 3). The long-term efficacy of benzoate and the outcome of benzoate-treated patients also deserve more studies in the future.

While LDL levels did not increase after sodium benzoate or placebo treatment, the levels in sertraline-treated patients increased from 96.8 mg/dL to 105.1 mg/dL (Table 5), lending support to the previous notion that treatment with SSRIs is commonly associated with hypercholesterolemia, especially increased LDL (Raeder et al., 2006; Chávez-Castillo 2018). In adult patients, long-term (2 months or longer) use of sertraline or paroxetine was associated with increased LDL values (Herran et al., 2006; Wei et al., 2009). Further, participants who used sertraline or other SSRIs were more likely to have high LDL levels compared with participants who did not use any psychotropic drugs (Raeder et al., 2006). In another study (Fjukstad et al., 2016), after controlling for potential confounders, the drug dose and serum concentration of certain SSRIs, such as sertraline, paroxetine, escitalopram, citalopram, or fluoxetine, was associated with increased LDL concentration. Longer-term studies are warranted to further investigate the effect of benzoate on lipid profiles.

An unanswered issue is the best dosing strategy of sodium benzoate for late-life depression. For safety concern, we titrated doses gradually. The final mean dose, 769.2 ± 239.1 mg/d, was similar to that (716.7 ± 182.6 mg/d) in patients with early-phase Alzheimer’s disease (Lin et al., 2014a) and that (622.0 ± 340.6 mg/d) in patients with BPSD (Lin et al., 2019). For patients with schizophrenia, 2000 mg/d of benzoate showed better efficacy than 1000 mg/d (Lin et al., 2018). Future studies are needed to explore whether a higher dose of benzoate can be more effective for late-life depression. On the other hand, the optimal dose of sertraline for geriatric patients also deserves more studies. However, at the doses used in the current study, sertraline recipients withdrew earlier, mainly due to adverse effects, than both benzoate receivers and placebo-treated patients (Fig. 1; Table 3).

This study was limited by its moderate sample size, which might have led to underpowered results. However, the sample size (n = 39) of each treatment group in the present study was similar to that (n = 30) of the previous study on sodium benzoate for the treatment of early-phase Alzheimer’s disease (Lin et al., 2014a). The second limitation is the 8-week treatment duration. It remains unclear whether a longer-term (such as 12 weeks) treatment of benzoate (or sertraline) can improve the depressive symptoms. Thirdly, whether the finding in Han Taiwanese can be extrapolated to other populations requires further studies. Finally, pro-inflammatory cytokines were reported to be higher in depressed patients (Zhang et al., 2017; Osimo et al., 2020) and in individuals with perceive stress (Jain et al., 2007; Casaletto et al., 2018). Further studies are warranted in exploring the potential roles of cytokines in the effects of benzoate among the patients with late-life depression.

## CONCLUSIONS

NMDAR enhancement, used here with sodium benzoate, shows promise as a novel mechanism for the drug development for late-life depression, especially for perceived stress symptom and cognitive dysfunction. Future studies with higher doses and longer treatment duration are warranted.

## Supplementary Material

pyac006_suppl_Supplementary_Data

## Data Availability

The data will be available by request approved by institutional review boards of both sites (applicants and owners of data).
